# A cross-sectional study investigating the impact of dietary vitamin D intake and fortification in Jordanian adults with type 2 diabetes

**DOI:** 10.29219/fnr.v70.11503

**Published:** 2026-08-31

**Authors:** Taha Rababah, Muhammad Al-U’datt, Ahmad Alsaad, Sana Gammoh, Khaleel Jawasreh, Ali Almajwal, Dunia AL Halees, Numan AL-Rayyan, Vaida Bartkutė-Norkūnienė, Huda Fish

**Affiliations:** 1 Department of Nutrition and Food Technology, Jordan University of Science and Technology, Irbid Jordan; 2 Physics Department, College of Science, Imam Mohammad Ibn Saud Islamic University (IMSIU), P.O. Box 90950, Riyadh 11623, Saudi Arabia; 3 Department of Animal Production, Faculty of Agriculture, Jordan University of Science and Technology, Irbid, Jordan; 4 Department of Community Health Sciences, College of Applied Medical Sciences, King Saud University, Riyadh, Saudi Arabia; 5 School of Medicine and Public Health, University of Wisconsin-Madison, Madison, WI, USA; 6 Faculty of Business and Technologies, Utena University of Applied Sciences, Utena, Lithuania; 7 School of Biological Science, Queen’s University Belfast,19 Chlorine Gardens, Belfast, Northern Ireland, BT9 5DL, United Kingdom

**Keywords:** vitamin D, type 2 diabetes mellitus, food fortification, nutritional value, chronic disease

## Abstract

**Background:**

Vitamin D is crucial in maintaining bone strength and supporting immune function and may also influence metabolic health, including glycemic control. This study aimed to investigate the relationship between the vitamin D intake and markers of glycemic health in Jordanian adults with type 2 diabetes mellitus (T2DM).

**Methods:**

This cross-sectional study was conducted on 240 patients with T2DM aged 42–69 years. Convenience sampling was used to recruit participants from hospitals across Jordan. Participants with conditions or medications impacting vitamin D status (e.g. celiac disease and antiepileptic drugs) were excluded from the study. Data on demographics, medical history, lifestyle, and dietary intake were collected using a structured questionnaire. Liquid chromatography with tandem mass spectrometry analysis was used to estimate the vitamin D content of commonly consumed foods. Serum 25(OH)D and glycated hemoglobin (HbA1c) levels were measured. Normality was tested using the Shapiro-Wilk test. Pearson’s correlation and analysis of variance (ANOVA) were used to determine links between the dietary vitamin D intake, serum vitamin D levels, and glycemic outcomes.

**Results:**

The mean dietary vitamin D intake was 150 ± 140 international units/day, with tuna, yogurt, pita bread, and non-fortified Ultra-High Temperature (UHT) milk as primary sources. Total dietary vitamin D intake showed no significant association with fasting blood glucose but was inversely correlated with HbA1c (*r* = –0.38, *P* < 0.05). Dairy (*r* = 0.42, *P* < 0.05) and fish (*r* = 0.35, *P* < 0.05) intakes were positively associated with serum 25(OH)D levels (mean: 21.3 ± 4.4 ng/mL), unlike meat, eggs, or bread. Higher income, education, and sun exposure were linked to higher levels of vitamin D in the serum. Vitamin D intake and status were found to be significantly associated with age, body mass index (BMI), frequency of breakfast consumption, and snack intake.

**Conclusion:**

Consuming vitamin D-rich sources, including dairy, fish, and fortified foods, was associated with higher serum vitamin D levels, which were related to better HbA1c levels. This suggests a potential benefit for glycemic control and broader diabetes management. A cross-sectional design makes it impossible to distinguish cause and effect, and factors such as supplement use or varying levels of food fortification complicate the interpretation of the results. Longer-term and intervention-based studies are necessary before a stronger claim can be made.

## Popular scientific summary

- Adults with type 2 diabetes may benefit from including vitamin D-rich foods, such as dairy and fish, in their diet. Higher intake of these foods is associated with higher vitamin D levels and better blood sugar control. Age, body weight, sun exposure, and lifestyle can also influence vitamin D status.
- Overall, these findings highlight the potential role of a vitamin-D-rich diet in supporting diabetes management.

Type 2 diabetes mellitus (T2DM) is a long-term metabolic disorder defined by elevated blood glucose levels, resulting from insufficient insulin production, reduced insulin sensitivity, or a combination of these factors (1). The global prevalence of T2DM is rising, driven by risk factors such as obesity (body mass index [BMI] ≥ 25 kg/m^2^), smoking, and sedentary lifestyles (2, 3). In Jordan, the prevalence of T2DM exceeds the global average, particularly among middle-aged and older adults, posing a significant public health challenge (4, 5). Lifestyle strategies such as regular physical activity and maintaining a healthy weight have consistently been shown to lower the risk of developing T2DM and to support better glycemic control (6).

Recent studies suggest that vitamin D status may be linked to the risk of developing T2DM. Vitamin D is present in two primary forms: ergocalciferol (D2), which is derived from plant-based sources, and cholecalciferol (D3), obtained either from animal-derived foods or produced endogenously in the skin following exposure to ultraviolet B (UVB) radiation (7). Both forms require hepatic and renal hydroxylation to make the active metabolite, 1,25-dihydroxyvitamin D (calcitriol) (7). Beyond its role in calcium and phosphorus homeostasis, calcitriol may enhance insulin secretion by stimulating pancreatic beta cells, regulating calcium influx, and improving insulin sensitivity via increased insulin receptor expression and reduced inflammation (8–11). Several observational studies have linked low-serum vitamin D levels to impaired glucose homeostasis. Although randomized controlled trials (RCTs) investigating the effects of vitamin D supplementation have produced mixed outcomes, some evidence indicates a beneficial effect in delaying the onset of T2DM among people with prediabetes (11–14). Consistent with this, a recent meta-analysis reported that vitamin D supplementation was associated with a 15% lower risk of developing T2DM in this high-risk group, suggesting its potential preventive value (11).

Jordan has a high prevalence of vitamin D deficiency, particularly among women, which is attributed to reduced sun exposure, traditional dress patterns, and inadequate consumption of vitamin D-rich foods (15–17). Fortified food intake remains low, and there is limited research assessing the influence of dietary vitamin D on glycemic control in individuals with T2DM (18). To address this gap, the current cross-sectional study examines the associations between the dietary vitamin D intake from natural and fortified sources and glycated hemoglobin (HbA1c) levels in Jordanian adults with T2DM. The specific aims are to:

1. determine vitamins D2 and D3 content in foods commonly consumed in Jordan using Liquid chromatography with tandem mass spectrometry (LC-MS/MS) analysis.
2. evaluate consumption patterns of vitamin D-containing foods among adults with T2DM using a validated dietary survey.
3. estimate total daily vitamin D intake based on food composition data and dietary surveys.
4. investigate associations between the dietary vitamin D intake, circulating 25(OH)D levels, and glycemic control (HbA1c), adjusting for confounding variables such as age, sex, BMI, sun exposure, and supplementation.

We propose that higher dietary vitamin D intake, particularly from sources like dairy and fish, is associated with improved serum vitamin D levels and better glycemic control. This study aims to evaluate the role of dietary vitamin D in the management of type 2 diabetes among Jordanians, to generate evidence that can guide and inform nutritional strategies for this high-risk population.

## Materials and methods

### Design and setting of the study

This cross-sectional study examined the relationship between the dietary vitamin D intake and glycemic control in individuals with T2DM at several government hospitals in both urban and rural areas of Jordan. Validated questionnaires, anthropometric measures, biochemical assays, and food sample analyses were used to gather data and determine the amount of vitamin D present.

### Participants and eligibility

A cohort of 240 Jordanian adults, aged 42–69 years and with confirmed T2DM, were recruited using convenience sampling at the outpatient clinics of government hospitals. This age range was selected due to the high prevalence of T2DM in this demographic (16). Inclusion criteria were as follows:

- Confirmed T2DM diagnosis
- Age 42–69 years
- Jordanian nationality
- Attendance at participating hospitals during the study period

Exclusion criteria included the following:

- Conditions affecting vitamin D metabolism (e.g. celiac disease, gallbladder disease, and thyroid disorders)
- Use of medications altering vitamin D levels (e.g. antiepileptic or anti-obesity drugs)

All participants received a standard 50,000 international units (IU) monthly vitamin D supplement (~1,666 IU/day). A written-informed consent was obtained from all participants, and the study received approval from the Institutional Review Board of [Jordan University of Science and Technology], in accordance with the ethical standards outlined in the Declaration of Helsinki.

### Data Collection

#### Survey questionnaire

A validated food frequency questionnaire (FFQ) was administered to all 240 participants to assess dietary habits, focusing on dietary intake from the frequency and portion sizes of vitamin D-rich foods (e.g. fish, dairy, and fortified products). The FFQ, pre-tested for reliability in a pilot sample of 20 participants, also captured data on lifestyle factors (e.g. sun exposure duration, smoking, and physical activity), medical history (e.g. insulin use), and sociodemographic characteristics (e.g. age, sex, education, income, skin color, and dress style).

#### Anthropometric measurements

Standardized protocols were employed to obtain anthropometric data. Participants’ body weight was recorded to the nearest 0.1 kg using a calibrated Tanita MC-780U scale, while height was measured to the nearest 0.1 cm with a wall-mounted stadiometer. BMI was calculated as weight in kilograms divided by height in meters squared (kg/m^2^).

#### Biochemical measurements

Venous blood samples were collected after an overnight fast and stored in serum separator tubes (for 25-hydroxyvitamin D [25(OH)D] and fasting blood glucose [FBG]) and ethylenediaminetetraacetic acid (EDTA) tubes (for HbA1c) at 2–8°C for immediate processing or –20°C for more extended storage. Serum 25(OH)D concentrations were measured using a chemiluminescent immunoassay (DiaSorin LIAISON 25 OH Vitamin D TOTAL Assay), HbA1c via high-performance liquid chromatography (HPLC), and FBG using standard enzymatic assays at the accredited hospital laboratories.

### Food sample collection and vitamin D analysis

#### Sample collection and preparation

Thirteen commonly consumed foods, identified via the FFQ, were purchased from local Jordanian markets. These included yogurt, semi-hard cheese, canned tuna (water-packed), raw chicken liver, raw beef liver, fortified chocolate soy milk, fortified powdered milk, non-fortified UHT milk, pita bread, canned mushrooms, egg yolk, and sardines. Fresh solid foods were homogenized by mincing or crushing to ensure representative subsampling.

#### Chemicals and standards

Analytical-grade chemicals (≥ 99% purity, Sigma-Aldrich, USA) were used, including vitamin D2 (ergocalciferol, Cat. No. E5750), vitamin D3 (cholecalciferol, Cat. No. C9756), methanol, ethanol, hexane, formic acid, and acetonitrile.

#### Vitamin D extraction

Vitamin D was extracted following a validated protocol (19). Briefly, 3.0 g of homogenized food sample was mixed with 3 mL of 39% ethanolic NaOH for saponification and incubated at 60°C for 40 min in a shaking water bath. A 1 mL internal standard (vitamin D2, except for mushrooms, where D3 was used) was added, followed by extraction with 12 mL hexane and centrifugation at 5,000 rpm for 5 min. The supernatant was evaporated under nitrogen and reconstituted with 500 μL of the mobile phase (methanol:acetonitrile:water, 68:30:2, with 0.1% formic acid).

#### LC-MS/MS analysis

Vitamin D content was quantified using an Atmospheric Pressure Ionization (API) 3200 LC-MS/MS system (Thermo Scientific) with a C18 Thermo Gold column (4.5 × 100 mm × 5 μm) (19). A 50 μL aliquot was injected, with a mobile phase flow rate of 1 mL/min. Calibration curves (0–1,000 ng/mL, *R*^2^ > 0.99) were prepared from 100 mg/L stock solutions of vitamins D2 and D3 in ethanol. The method was validated for accuracy, precision, linearity, and sensitivity using quality control samples.

### Statistical analysis

Statistical analyses were carried out using SPSS version 22. Normality of continuous data was examined using the Shapiro–Wilk test, and the Levene’s test was used to verify homogeneity of variances. Variables that followed a normal distribution, such as serum 25(OH)D and BMI, were expressed as mean ± standard deviation (SD). Group differences in continuous variables were analyzed using one-way analysis of variance (ANOVA). Pearson’s correlation coefficients were estimated to examine associations between the vitamin D intake (dietary and serum levels) and diabetes-related biomarkers, including HbA1c and FBG. These analyses were adjusted for potential confounders, including age, BMI, education level, and income. Results were deemed statistically significant if the *P*-value was < 0.05.

### Bias mitigation

Convenience sampling may introduce selection bias, which can be mitigated by recruiting from multiple hospitals across Jordan. Variability in biochemical measurements was minimized by using accredited laboratory assays. The uniform 50,000 IU monthly vitamin D supplementation among participants was accounted for by including total vitamin D intake (dietary + supplemental) as a covariate in statistical analyses.

## Results

### Participant characteristics

This section presents the baseline characteristics of 240 Jordanian adults with T2DM, aged 42–69 years, recruited from government hospitals in Jordan. All participants received a standard 50,000 IU monthly vitamin D supplement (~1,666 IU/day). Table 1 summarizes demographic, clinical, socioeconomic, and lifestyle characteristics, reported as *n* (%), mean ± SD.

**Table 1:** Baseline characteristics and variables of Jordanian T2DM patients (aged 42–69 years), where the results are presented as a mean value or (%)

Variables	*n* (%)
Gender	
Male	96 (40)
Female	144 (60.0)
Age	
40–50	78 (32.5)
51–60	90 (37.5)
60>	72 (30.0)
BMI (kg/m^2^)	31.8 ± 5.4
Obese (BMI >30)	157 (65.4%)
HbA1c (%)	8.2 ± 1.5
Fasting glucose (mg/dL)	165 (135–210)
Education levels	
Illiterate	6 (2.5)
Primary school	54 (22.5)
Secondary	60 (25.0)
Bachelor	102 (42.5)
Master or PhD	18 (7.5)
Family income (JD)	
Less than 200	48 (20.0)
200–500	126 (52.2)
501–2,000	60 (25.0)
More than 2,000	6 (2.5)
Social status	
Married	210 (87.5)
Single	18 (7.5)
Other	12 (5.0)
Skin color	
Light	127 (53)
Dark	112 (47)
Dress style	
Head cover	154 (64.2)
Gloves	10 (4.2)
Face cover	5 (2.1)
No cover	86 (35.8)
Vitamin D intake & supplementation	
Dietary vitamin D intake (IU/day)	150 ± 140
Monthly vitamin D supplement	100% (50,000 IU/month, ~1,666 IU/day)

Note: Data are presented as *n* (%) for categorical variables, mean ± SD for normally distributed continuous variables. T2DM: Type 2 Diabetes Mellitus; BMI: body mass index; HbA1c: glycated hemoglobin; IU: international units; SD: standard deviation.

#### Demographic and clinical characteristics

The cohort consisted of 60.0% females (*n* = 144) and 40.0% males (*n* = 96), which is consistent with the higher female participation in Jordanian T2DM studies (20). The mean age was 55.3 ± 6.9 years, with a distribution of 32.5% (*n* = 78) aged 40–50 years, 37.5% (*n* = 90) aged 51–60 years, and 30.0% (*n* = 72) aged over 60 years. Clinically, 65.4% (*n* = 157) were obese (BMI >30 kg/m^2^), with a mean BMI of 31.8 ± 5.4 kg/m^2^. Mean HbA1c was 8.2 ± 1.5%, demonstrating inadequate glycemic control.

#### Socioeconomic characteristics

Most participants (87.5%, *n* = 210) were married, 7.5% (*n* = 18) were single, and 5.0% (*n* = 12) were in other marital statuses. Education levels varied: 42.5% (*n* = 102) held a bachelor’s degree, aligning with prior findings (16), 25.0% (*n* = 60) completed secondary school, 22.5% (*n* = 54) primary school, 7.5% (*n* = 18) had a master’s or PhD, and 2.5% (*n* = 6) were illiterate. Family income was predominantly moderate, with 52.5% (*n* = 126) earning 200–500 JD/month, 25.0% (*n* = 60) earning 501–2,000 JD/month, 20.0% (*n* = 48) earning < 200 JD/month, and 2.5% (*n* = 6) earning > 2,000 JD/month.

#### Lifestyle and vitamin D-related factors

Skin color, which influences vitamin D synthesis due to melanin’s absorption of UVB radiation (19, 21), was light in 53.0% (*n* = 127) and dark in 47.0% (*n* = 112). For dress style, affecting sun exposure, 64.2% (*n* = 154) were wearing head covers, 4.2% (*n* = 10) were wearing gloves, 2.1% (*n* = 5) were wearing face covers, and 35.8% (*n* = 86) were wearing no cover. These proportions are lower than in studies with predominantly female cohorts (e.g. 85.5% head cover (15)), likely due to higher male participation. Cultural dress practices (e.g. hijab and niqab) are associated with vitamin D deficiency in Arab populations (22–28). Mean dietary vitamin D intake was 150 ± 140 IU/day, lower than the 309 IU/day reported elsewhere (29), which included supplemental intake beyond dietary sources. The low dietary intake, combined with limited sun exposure, highlights the reliance on supplementation in this cohort.

### Clinical and anthropometric characteristics

This section presents the clinical and anthropometric characteristics of the study sample, which included 240 Jordanian diabetic patients aged 42–69 years. The observational study characteristics, including participant demographics, clinical parameters, and medication use, are summarized in Table 2.

**Table 2:** Clinical and anthropometric characteristics of Jordanian diabetic patients aged 42–69 years (*n* = 240)

Variables	*n*	%
Body mass index (BMI)		
Normal (< 25 kg/m^2^)	30	12.5
Overweight (25–29.9 kg/m^2^)	72	30.0
Obesity (≥ 30 kg/m^2^)	138	57.5
Medication use in the last 3 months		
Antihypertensive drugs	156	65
Lipid-lowering drugs	120	50
Insulin intake	90	37
Diabetic agent drugs	240	100
Mean ± SD		
Vitamin D blood levels	240*	22.7 ± 4.48^$^
HbA1c blood levels	240	7.4 ± 1.5^$^

Data are presented as percentages or as mean ± standard deviation.

$ Mean ± standard deviation.

* Vitamin level (< 20 ng/mL (deficiency); 20–30 (inadequate); > 30 (Adequate) (21) HbA1c < 7% indicates optimal glycemic control per American Diabetes Association (ADA) guidelines. HbA1c: glycated hemoglobin; SD: standard deviation.

#### Anthropometric characteristics

The mean BMI was 31.6 ± 5.2 kg/m^2^, indicating obesity (BMI ≥ 30 kg/m^2^) per (30). Participants were distributed as follows: 12.5% (*n* = 30) normal weight (BMI 18.5–24.9 kg/m^2^), 30.0% (*n* = 72) overweight (BMI 25.0–29.9 kg/m^2^), and 57.5% (*n* = 138) obese (BMI ≥ 30 kg/m^2^). ANOVA test confirmed significant differences between BMI categories (*P* < 0.05), with the obese group differing significantly from the normal weight and overweight groups. This high obesity prevalence aligns with regional studies reporting elevated BMI in Middle Eastern diabetic populations. The mean BMI exceeds the 27.9 kg/m^2^ reported by (31), possibly due to lifestyle factors or sample-specific characteristics.

#### Clinical characteristics

Medication use was prevalent, with 65% (*n* = 156) of participants using antihypertensive drugs and 50% (*n* = 120) using lipid-lowering drugs, reflecting high rates of hypertension and dyslipidemia. These figures are notably higher than those reported by (32), who noted 42% and 9% prevalence for hypertension and dyslipidemia, respectively. Insulin use was reported by 37% (*n* = 90) of participants comparable to the 39.2% reported in (33). All participants (100%, *n* = 240) were on diabetic medications, consistent with the study’s focus on diabetic patients.

The mean serum vitamin D (25(OH)D) level was (22.7 ± 4.48 ng/mL), which falls within the ‘inadequate’ range (20–30 ng/mL) as per (15). This is slightly higher than the 20.16 ng/mL reported by (33), suggesting regional variations in vitamin D status.

The mean HbA1c was 7.4 ± 1.2%, demonstrating suboptimal glycemic control among the study population (< 7% considered optimal per ADA guidelines). This is lower than the 8.94% reported by (33), suggesting relatively better glycemic management in this cohort. These findings reflect the coexistence of obesity, poor glycemic outcomes, and suboptimal vitamin D status frequently observed in Middle Eastern populations with type 2 diabetes.

To conclude, the high prevalence of obesity (57.5%) and overweight (30.0%) emphasizes the dual burden of obesity and diabetes in this population, consistent with regional trends (16). The frequent use of antihypertensive and lipid-lowering medications highlights the burden of comorbidity, necessitating integrated management strategies. The suboptimal HbA1c levels (7.4%) and inadequate vitamin D levels (22.7 ng/mL) suggest areas for clinical intervention, aligning with previous findings on poor glycemic control and vitamin D insufficiency in Middle Eastern diabetic cohorts (33).

### Dietary habits and meal frequency

**Fig. 1:**
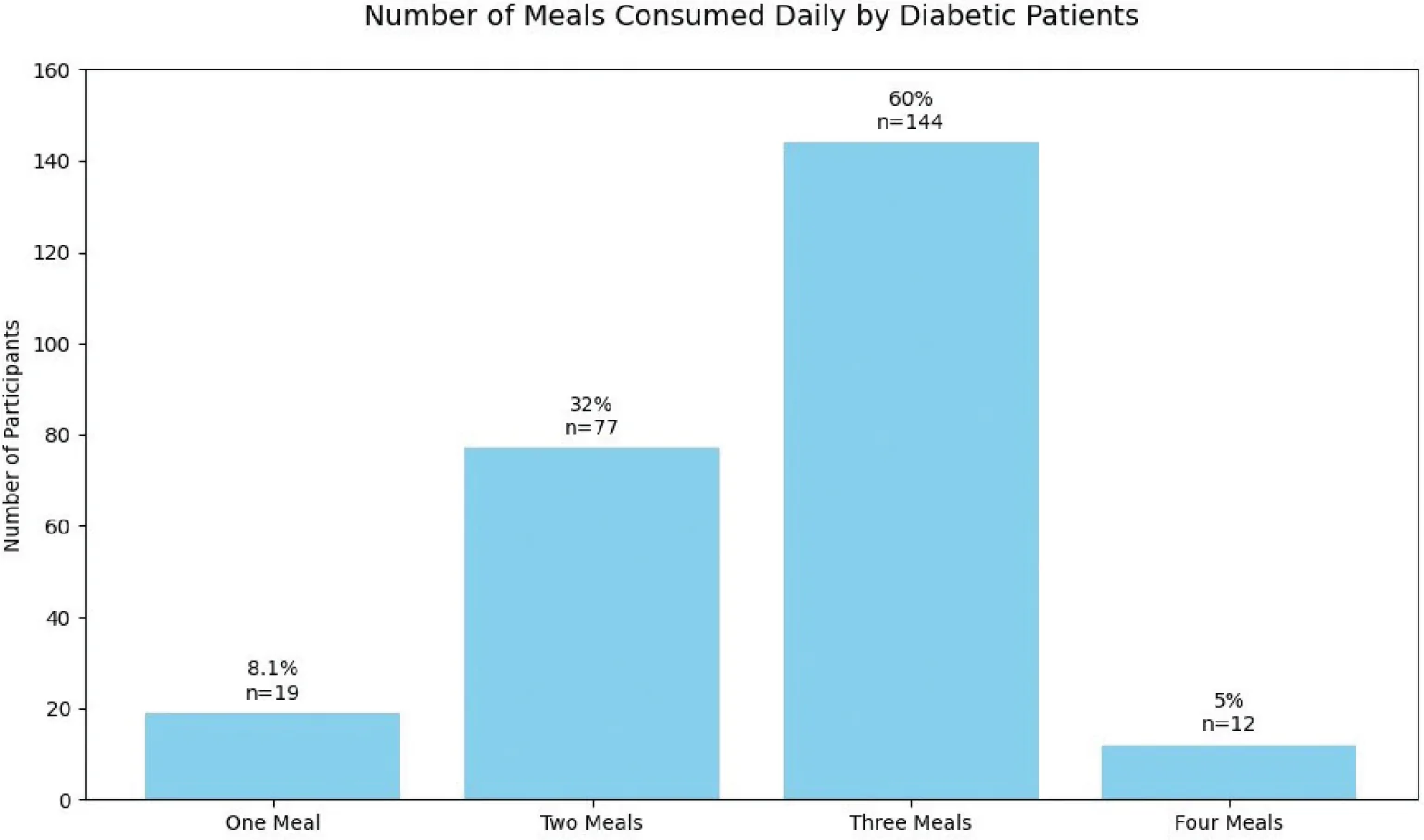
Daily meal frequency among Jordanian diabetic patients (aged 42–69 years). Data are presented as percentages (%) for the participants consuming 1, 2, 3, or 4 meals daily.

The dietary habits and meal frequency of 240 Jordanian diabetic patients aged 42–69 years, with a focus on their impact on vitamin D intake, are presented in Fig. 1 and Table 3.

**Table 3:** Effect of dietary habits on vitamin D intake (IU) among Jordanian diabetic patients (aged 42–69 years)

Variables and dietary habit	Vitamin D intake (IU)*	*P*
Number of meals per day		0.26
One meal	54.1 ± 1.6	
Two meals	139.91 ± 128.3	
Three meals	160.43 ± 151.2	
More than three	154.46 ± 92.7	
Breakfast intake & frequency		**0.0**
Daily	377.0 ± 4.1^a^	
3–4 times a week	151.88 ± 140.2^b^	
Rarely	62.57 ± 47.1^c^	
Number of snacks & frequency per day		**0.03**
One	112.48 ± 100.1^c^	
Two	154.51 ± 149.1^b^	
More than three	183.28 ± 146.3^a^	

Values are presented as mean ± SD – values determined with ANOVA. Statistical comparisons were performed using ANOVA. Superscript letters indicate significant differences between groups; values sharing the same letter are not significantly different. Bold *P*-values denote statistical significance at *P* < 0.05. Breakfast and snack frequency data are based on self-reported dietary habits over the past 3 months.

* IU: International Units; ANOVA: analysis of variance; SD: standard deviation.

#### Meal frequency

The number of meals consumed daily is shown in Fig. 1. Most participants reported three meals per day (60%; *n* = 144), followed by two meals (32%; *n* = 77), one meal (8.1%; *n* = 19), and four meals (5%; *n* = 12). From a nutritional perspective, consuming one or two meals per day may restrict dietary diversity and nutrient intake. In contrast, three or more meals daily are generally recommended to support adequate micronutrient intake (including vitamin D), promote dietary variety, and maintain metabolic health. These findings align with previous research highlighting the benefits of regular meal distribution for glycemic control in individuals with diabetes (34).

#### Effect of dietary habits on vitamin D intake

Table 3 summarizes the association between dietary habits (meal frequency, breakfast consumption, and snack frequency) and vitamin D intake, measured in IUs. Statistical analyses were conducted using ANOVA.

As shown in Table 3, mean vitamin D intake varied across meal frequencies: 54.1 ± 30.2 IU for those consuming one meal per day, 139.9 ± 128.3 IU for two meals, 160.4 ± 151.2 IU for three meals, and 154.5 ± 92.7 IU for four meals. However, the differences were not statistically significant (*P* = 0.26, *P* > 0.05). This lack of significance may be due to limited dietary sources of vitamin D in the consumed meals or variability in portion sizes; no prior studies have directly examined this relationship in diabetic populations.

Breakfast consumption was significantly associated with vitamin D intake. Participants who consumed breakfast daily had the highest mean vitamin D intake (377.0 ± 41.0 IU), compared to those consuming breakfast 3–4 times per week (151.8 ± 140.2 IU) or rarely (62.5 ± 47.0 IU). These findings indicate that breakfast frequency has a significant influence on vitamin D intake (*P* < 0.05). Regular breakfast consumption, particularly of fortified cereals, may present a key opportunity to enhance vitamin D intake in this population, consistent with (35), who reported higher intakes of vitamin D among Canadians who regularly consume breakfast cereals.

Snack frequency was significantly associated with vitamin D intake (*P* < 0.05). Participants consuming one snack per day had a mean vitamin D intake of 112.5 ± 100.1 IU, compared to 154.5 ± 149.0 IU for those consuming two snacks, and 183.3 ± 146.3 IU for those consuming three or more snacks per day. This suggests that more frequent snacking may increase opportunities for consuming vitamin D-rich foods, such as fortified snacks, potentially driven by higher appetite, greater dietary variety, and a higher overall food intake.

In conclusion, the predominance of three meals per day (60%) supports dietary recommendations for diabetic patients to maintain metabolic stability. The significant association between breakfast frequency and vitamin D underscores the importance of regular breakfast consumption, which may be attributed to the inclusion of fortified foods. Similarly, higher snack frequency correlated with increased vitamin D intake, suggesting that snacks may contribute to overall nutrient intake. The lack of significance for meal frequency may warrant further investigation into specific food choices and portion sizes. These findings underscore the importance of targeted dietary interventions to enhance vitamin D intake in diabetic populations, particularly by modifying breakfast and snacking behaviors.

### Effect of age and BMI on vitamin D intake

#### Age and vitamin D intake

A significant correlation was observed between age and vitamin D intake (*P* = 0.04), as shown in Table 4. The lower intake (126.1 ± 123.8 IU) in the 51–60 age group compared to the 40–50 age group (151.2 ± 135.4 IU) may be attributed to reduced appetite or changes in dietary habits and food consumption, as supported by (36, 37). The higher intake in participants over 60 years (181.3 ± 159.9 IU) could potentially reflect the increased consumption of vitamin D-fortified foods, such as milk, which is common in older populations to support bone health. These findings are consistent with studies indicating age-related variations in nutrient intake due to physiological and behavioral factors (36, 37).

**Table 4:** Correlation of vitamin D intake with age and body mass index among Jordanian diabetic patients

Age group	Vitamin D intake (IU, Mean ± SD)
40–50 years	151.23 ± 135.40^ab^
51–60 years	126.05 ± 123.80^b^
60 > years	181.34 ± 159.90^a^
*P*-value	**0.04**
**Halves of vitamin D intake (IU)**	**Body mass index (BMI, kg/m^2^)**
Vitamin D intake > 400 IU/day	31.31^a^ ± 5.9
Vitamin D intake< 400 IU/day	33.03^b^ ± 5.1
*P*	**0.02**

All values are presented as mean ± SD. Comparisons were conducted using ANOVA. Superscript letters (^a, b^) indicate significant differences between groups; values sharing the same letter are not significantly different. Bold *P*-values indicate statistical significance at *P* < 0.05. IU: International Units; ANOVA: analysis of variance; SD: standard deviation.

#### BMI and vitamin D intake

A significant association was found between the vitamin D intake and BMI (*P* = 0.02), which was previously reported by (38). Participants consuming > 400 IU/day of vitamin D had a lower mean BMI (31.3 ± 5.9 kg/m^2^) compared to those consuming less than < 400 IU/day (33.0 ± 5.1 kg/m^2^). This inverse relationship aligns with research by (39), which reported that higher vitamin D intake promotes fat cell apoptosis through calcium signaling and the calcium/calpain/caspase-dependent pathway, potentially leading to a reduction in BMI. Additionally, higher BMI is strongly linked to increased risk of type 2 diabetes due to visceral fat accumulation, which induces insulin resistance via elevated free fatty acid (FFA) delivery to the liver and impaired insulin-mediated glucose uptake in skeletal muscle (3, 40).

The lipolysis of triglycerides caused by significant visceral fat releases FFAs straight into the portal vein, where they are subsequently delivered to the liver, resulting in insulin resistance (41). Insulin’s capacity to reduce hepatic glucose synthesis is weakened by increased FFA delivery to the liver, and increased FFA concentration prevents insulin-facilitated glucose entry in skeletal muscle (42). These findings further highlight the multifactorial role of nutritional factors, such as vitamin D intake and anthropometric measures, especially BMI, in the management of type 2 diabetes.

This significant correlation between age and the vitamin D intake highlights age-specific dietary patterns, with older adults (over 60 years) potentially benefiting from consuming foods that are fortified with vitamin D. The inverse relationship between the vitamin D intake and age is notable. BMI suggests a role for vitamin D in fat metabolism, which may have implications for diabetes management, given the link between visceral fat, insulin resistance, and type 2 diabetes. These findings underscore the importance of targeted nutritional interventions to optimize vitamin D intake, particularly in middle-aged adults (51–60 years) with lower intakes, in support of metabolic health and the mitigation of BMI-related complications.

### Sociodemographic factors and serum vitamin D levels

Table 5 illustrates the associations between sociodemographic characteristics and serum 25(OH)D levels among Jordanian adults with type 2 diabetes.

**Table 5:** Effect of sociodemographic status on serum vitamin D levels among Jordanian diabetic patients

Variables	Vitamin D blood level (ng/mL, Mean ± SD)	*P*
Age group		0.441
40–50 years	23.12 ± 5.6	
51–60 years	21.14 ± 3.0	
60 > years	22.39 ± 4.4	
Education levels		**0.000**
Illiterate	20.83 ± 0.41^b^	
Primary school	22.96 ± 3.71^a^	
Secondary	22.25 ± 4.8^a^	
Bachelor	21.84 ± 4.9^a^	
Master or PhD	23.33 ± 1.2^a^	
Family income (JD)		**0.000**
< 200	21.68 ± 4.3^b^	
200–500	21.00 ± 5.1^b^	
501–2,000	20.20 ± 5.9^b^	
> 2,000	22.75 ± 4.5^a^	
Sun exposure (daily)		**0.000**
> 15 min exposure	25.10 ± 3.71^a^	
< 15 min exposure	19.31 ± 3.16^b^	

Values are presented as mean ± SD – values determined with ANOVA. Statistical comparisons were performed using ANOVA. Superscript letters indicate significant differences between groups; values sharing the same letter are not significantly different. Bold *P*-values denote statistical significance at *P* < 0.05. ANOVA: analysis of variance; SD: standard deviation.

#### Age and serum vitamin D levels

Serum vitamin D levels did not show a significant correlation with age (*P* = 0.44). Mean serum vitamin D levels were 23.1 ± 5.6 ng/mL (40–50 years), 21.1 ± 3.0 ng/mL (51–60 years), and 22.4 ± 4.4 ng/mL (>60 years). While previous studies in non-diabetic populations have reported age-related declines in vitamin D levels (43), this discrepancy may be attributed to factors specific to individuals with type 2 diabetes, such as medication use, comorbidities, dietary intake, supplementation habits, or possibly reduced skin synthesis. Certain statins have been associated with increased serum vitamin D levels in older adults (44). These factors may help explain the lack of a significant age-related trend in the present study.

#### Education level, income, and serum vitamin D levels

Serum vitamin D levels and educational levels were significantly correlated (*P* < 0.001). Participants with higher education (primary school or above) had significantly higher mean vitamin D levels (22.0–23.3 ng/mL) compared to illiterate participants (20.8 ± 0.4 ng/mL). This aligns with findings by (45), suggesting that higher education may be associated with a greater awareness of healthy dietary practices and the importance of consuming vitamin D-rich foods or taking vitamin D supplements. Similarly, individuals with household incomes above 2,000 JD/month had higher mean vitamin D levels (22.8 ± 4.5 ng/mL) than those earning less than 500 JD/month (range: 20.2–21.7 ng/mL). These differences may reflect disparities in health literacy, dietary diversity, and access to supplements and vitamin D-rich foods, such as fortified products and fish, which will impact vitamin D status (45).

#### Sun exposure and serum vitamin D levels

Sun exposure has a significant influence on serum vitamin D levels (*P* < 0.001). Participants with >15 min of daily sun exposure had significantly higher serum 25(OH)D levels and the mean vitamin D level of (25.1 ± 3.7 ng/mL), compared to (19.3 ± 3.2 ng/mL) for those with 15 min of sun exposure. This finding is supported by a prior regional study (46), which emphasized the role of adequate sun exposure in promoting cutaneous vitamin D synthesis, a critical factor in populations with limited dietary vitamin D intake.

### Vitamin D content

The concentrations of vitamins D2 and D3 in selected food items were measured using liquid chromatography-tandem mass spectrometry (LC-MS/MS, API 3200 model), with results converted from ng/mL to μg/100 g. These results provide insights into the vitamin D content of commonly consumed foods in Jordan and their potential contribution to dietary vitamin D intake among diabetic patients aged 42–69 years.

#### Vitamin D concentrations of selected commonly consumed Jordanian food items

Table 6 details the mean concentrations of vitamins D3 and D2 in various food items. Among the tested foods, canned tuna (in water) exhibited the highest vitamin D3 concentration (1.65 ± 0.83 μg/100 g), followed by yogurt (0.18 ± 0.10 μg/100 g), fortified pita bread (0.17 ± 0.23 μg/100 g), and plain liquid UHT milk (0.10 ± 0.20 μg/100 g).

**Table 6:** Concentrations of vitamins D_3_ and D_2_ in some selected food items

Food item	Vitamin D3 (μg/100 g, Mean ± SD)*
Plain liquid milk	0.10 ± 0.2^c^
Powder milk^$^	0.03 ± 0.52^de^
Semi-hard cheese	0.07 ± 0.05^cd^
Fish (tuna)	1.65 ± 0.83^a^
Pita bread^$^	0.17 ± 0.23^b^
Yogurt	0.18 ± 0.1^b^
Lamb liver	0.0 08 ± 0.01^e^
Chicken liver	0.02 ± 0.04^de^

**Food item**	**Vitamin D_2_ (μg/100 g)**

Soy milk^$^	0.061 ± 0.05^a^
Mushrooms	0.04 ± 0.01^b^

$ Fortified food item.

* Mean ± standard deviation.

Values with the same letters are not significantly different (*P* ≤ 0.05). SD: standard deviation.

Lower concentrations were observed in semi-hard cheese (0.07 ± 0.05 μg/100 g), fortified powdered milk (0.03 ± 0.52 μg/100 g), fortified, chicken liver (0.02 ± 0.04 μg/100 g), and lamb liver (0.008 ± 0.01 μg/100 g). No significant differences in vitamin D_3_ content were found among powdered milk, semi-hard cheese, chicken liver, and lamb liver (*P* > 0.05). For vitamin D2, fortified soy milk had the highest concentration (0.06 ± 0.05 μg/100 g), followed by mushrooms (0.04 ± 0.01 μg/100 g). Liver samples and powdered milk had trace levels of vitamin D_2_, consistent with global studies (47). The relatively low concentrations in dairy products (e.g. yogurt and milk) may reflect limited fortification practices in Jordan compared to other countries.

To our knowledge, no previous studies have reported the vitamin D content of soy milk. Our analysis found a value of 0.06 μg/100 g, which is higher than the American reported value of 0 μg/100 g (48), but lower than the Swiss value of 0.18 μg/100 g.

Vitamin D2 in Jordanian mushrooms (0.04 μg/100 g) had lower concentrations than Canadian (29.50 μg/100 g) and American (2.00 μg/100 g) mushrooms (48) but were higher than in France, Sweden, and Switzerland (0.00 μg/100 g). In mushrooms, vitamin D_2_ levels are primarily determined by ultraviolet (UV) exposure, which varies with cultivation practices, handling, processing methods (e.g. fresh vs. canned), and the mushroom variety (48–50). This reflects their unique ability to convert ergosterol to vitamin D_2_ when exposed to UV light (50).

#### Vitamin D content comparison with international data

Table 7 compares the vitamins D3 and D2 concentrations in Jordanian foods with those reported in international databases (Canada, Sweden, France, Turkey, USA, and Switzerland).

**Table 7:** Comparison of vitamins D3 and D2 concentrations (μg/100 g) in Jordanian foods with data from international studies

Food item (vitamin D3)	Canada	Sweden	France	Turkey	USA	Jordan
Fish (canned tuna)	1.20	4.20	4.80	1.1	2.0	1.65
Plain liquid milk (cow)	0.90	0.29	0.30	1.0	0.05	0.10
Yogurt (plain)	Traces	0.06	0.10	1.1	0.05	0.18
White hard cheese	0.70	0.18	0.63	0	0.65	0.07
Bread	0.00	0.46	<5	ND	0.00	0.17
Lamb liver	0.50	0.50	ND	ND	0.00	0.008
Chicken liver	0.00	0.40	0	ND	0.00	0.02

**Food item (vitamin D2)**	**Canada**	**Sweden**	**France**	**Switzerland**	**USA**	**Jordan**

Mushrooms	29.5	0.00	0.00	0.00	0.20	0.04
Soy milk	ND	ND	ND	1.80	0.00	0.06

Canada/(54), France/(63), Turkey/(64), USA/(48), Sweden/(58), Swiss/(65).

### Seafood

The vitamin D3 concentration in Jordanian canned tuna is 1.65 μg/100 g and was higher than in Canada (1.20 μg/100 g) and Turkey (1.10 μg/100 g) but lower than in France (4.80 μg/100 g), Sweden (4.20 μg/100 g), and the USA (2.00 μg/100 g). Variations in vitamin D3 content in fish, such as tuna, are influenced by species, geographic location, and dietary patterns (51).

In the United States, farmed salmon is a popular dietary source of fish; however, it contains only about one-quarter of the vitamin D3 present in wild Alaskan salmon. Some farmed salmon have also been reported to contain vitamin D2, as verified through LC-MS/MS testing (52). The high vitamin D3 content in canned tuna positions it as a key dietary source for these diabetic patients.

### Dairy products

**Plain liquid milk:** The vitamin D3 content in Jordanian plain liquid milk was measured at 0.10 ± 0.20 μg/100 g, which is higher than values reported for American milk (0.05 μg/100 g; (48)) and significantly higher than findings by (53) (0.002 μg/100 g). However, it is lower than levels in Swedish (0.29 μg/100 g), French (0.35 μg/100 g), Canadian (0.90 μg/100 g), and Turkish (1.00 μg/100 g) milk. These variations are likely due to differences in fortification practices, with Canada’s mandatory fortification (54) and Turkey’s active fortification policies contributing to higher levels. Additional factors influencing vitamin D3 content include livestock feed, supplementation, sunlight exposure, seasonal changes, and cow breeds (55).

**Powdered milk:** The vitamin D3 content in the tested powdered milk sample was 0.03 ± 0.52 μg/100 g, significantly lower than the labeled fortified value of 230 IU/100 g (equivalent to 5.75 μg/100 g). This discrepancy aligns with (55), which reported 0.25 μg/100 g for unfortified powdered milk and 11.5 μg/100 g for fortified samples. No central food database, including Table 7, provides vitamin D values for powdered milk. In Canada and the U.S., fortification of powdered milk, evaporated milk, and goat’s milk is mandatory under strict regulatory standards (56).

**Yogurt:** The vitamin D3 concentration in Jordanian yogurt was measured at 0.18 ± 0.10 μg/100 g, surpassing levels found in the U.S., France, Canada, and Sweden (all < 0.02 μg/100 g) (57). However, it was lower than that of Turkish yogurt (1.10 μg/100 g), where higher values are likely due to fortification or differences in the vitamin D content of the milk used. Overall, these reported differences are potentially linked to livestock diet, UV exposure, seasonality, and breed (55).

**White hard cheese:** The vitamin D_3_ content of white hard cheese varied considerably across countries, reflecting differences in fortification practices, production methods, and regulatory standards. The highest levels were reported in Canada (0.70 μg/100 g), the USA (0.65 μg/100 g), and France (0.63 μg/100 g), all of which are consistent with the use of fortification or differences in dairy production systems. Sweden reported a lower value (0.18 μg/100 g), while Jordan (0.07 μg/100 g) and Turkey (0 μg/100 g) showed negligible levels of vitamin D_3_ content. These differences likely reflect fortification policies, feed practices, sunlight exposure, and database methods. In Jordan, the low vitamin D_3_ levels in dairy products suggest limited fortification, which may contribute to poor vitamin D status in diabetic groups (mean serum vitamin D level: 22.7 ng/mL).

### Bread

Fortified Arabic pita bread in Jordan exhibited a vitamin D3 concentration of 0.17 μg/100 g, higher than the American (48) and Canadian bread (0 μg/100 g (54)) but lower than the Swedish bread (0.46 μg/100 g), likely due to the inclusion of vitamin D-rich ingredients like milk or eggs (58). In Jordan, Mowahad wheat flour, which constitutes over 90% of wheat flour consumption, has been fortified with iron and folic acid since 2002 (59, 60) and vitamin D at 550 IU/kg since 2010 (61). The wheat flour fortification program in Jordan is administered by the Ministry of Health’s Nutrition Division. In partnership with other government agencies, the division is responsible for setting fortification guidelines, procuring and distributing the micronutrient premix, and overseeing compliance and monitoring activities (60).

Despite this mandate, only trace levels of vitamin D were detected in bread, possibly due to inconsistent micronutrient premix supply or logistical procurement issues. Variations in vitamin D content across countries perhaps resulted from differences in fortification policies, production methods, livestock feed, seasonal sun exposure, and product labeling. The limited presence of vitamin D in Jordanian bread emphasizes the need for improved fortification consistency, enhanced public health strategies, and better consumer education to address the scarcity of natural and fortified dietary vitamin D sources. Remarkably, Arabic pita bread is not included in international food composition tables.

### Liver

**Lamb liver:** The vitamin D3 content in lamb liver was 0.008 μg/100 g, which is lower than Canadian and Swedish values (0.5 μg/100 g) but higher than American beef liver (0 μg/100 g), with no prior studies specifically reporting vitamin D in lamb liver.

**Chicken liver:** The vitamin D3 concentration in chicken liver is 0.025 μg/100 g, which exceeds the levels in the USA, France, and Canada (0 μg/100 g) but falling below Swedish level (0.4 μg/100 g) and (62) level (0.2 μg/100 g).

These variations in liver vitamin D levels are attributed to differences in species, fat content, environmental conditions, and seasonal factors (51). The liver’s critical role in vitamin D metabolism, converting it to 25-hydroxyvitamin D3, further influences these levels (63).

### Plant-based foods

**Mushrooms:** The vitamin D2 concentration in Jordanian mushrooms was 0.04 μg/100 g, which is significantly lower than Canadian (29.5 μg/100 g), American (0.2 μg/100 g), and canned white mushrooms (0.3 μg/100 g (49)) but higher than French, Swedish, and Swiss values (0 μg/100 g). These variations, as mentioned in the previous session, stem from differences in mushroom species and UV light exposure, which is critical for vitamin D2 synthesis (50, 54).

**Fortified soy milk:** The vitamin D2 concentration in Jordanian fortified soy milk was 0.06 ± 0.05 μg/100 g, which is higher than the U.S. value (0 μg/100 g) but lower than the Swiss value (0.18 μg/100 g). As highlighted earlier, there are no prior local studies that report the vitamin D content in soy milk.

#### Implications

Various factors, including fortification policies, production methods, livestock feed, sunlight exposure, and labeling practices, influence discrepancies in vitamin D content across countries. The limited natural and fortified dietary sources of vitamin D in Jordan highlight the need for enhanced fortification strategies, consistent micronutrient premix supply, and dietary recommendations to improve vitamin D intake in diabetic populations.

### Association between the total vitamin D intake and glycemic control (HbA1c)

Total daily vitamin D intake was estimated by multiplying the portion size of each food item by its reported frequency of consumption and vitamin D content and then summing the values across all foods (66). Based on intake levels, participants were categorized into two groups: those consuming ≤ 400 IU/day (*n* = 180) and those consuming > 400 IU/day (*n* = 60), as presented in Table 8. A significant association was observed between the vitamin D intake and HbA1c levels (*P* < 0.001, ANOVA adjusted for age, sun exposure, and insulin intake), with mean HbA1c levels of 7.50% for the ≤ 400 IU/day group and 6.91% for the > 400 IU/day group, indicating better improved long-term glycemic control with higher vitamin D intake. No significant association was found with fasting blood sugar (FBS) levels (*P* = 0.20), with means of 193.7 mg/dL (≤ 400 IU/day group) and 181.3 mg/dL (> 400 IU/day group). Higher vitamin D intake is associated with improved long-term glycemic control in Jordanian adults with type 2 diabetes, as evidenced by lower HbA1c levels, consistent with (33). The observed effect may be partly attributed to reduced insulin resistance, mediated through vitamin D receptors (VDRs) located in pancreatic β-cells as well as vitamin D-dependent calcium-binding proteins that facilitate insulin release (9). Additionally, vitamin D may enhance insulin sensitivity by stimulating the transcription of insulin receptor messenger ribonucleic acid (mRNA) (67).

**Table 8:** The effect of total vitamin D intake (IU) on HbA1c and fasting blood glucose levels among Jordanian diabetic patients aged 42–69 years

Variables	HbA1c %	FBS mg/dL
Total vitamin D daily intake		
≤ 400	7.50 ± 1.61^a^	193.71 ± 88.21^a^
> 400	6.91 ± 1.1^b^	181.31 ± 95.6^a^
*P*	**0.000**	0.2

All values are mean ± SD – values determined with ANOVA after adjustment for age, sun exposure, and insulin intake. Values in boldface indicate statistical significance (*P* < 0.05). Values with the same letters do not have a significant difference. Patients have received a monthly supplement of 50,000 IU of Vitamin D. HbA1c: Glycated hemoglobin; FBS: Fasting blood sugar; IU: International units; ANOVA: analysis of variance; SD: standard deviation.

### The effect of vitamin D intake from different food groups on serum vitamin D and HbA1c

As shown in Table 9, statistical analyses were adjusted for potential confounders, including sun exposure, sunblock use, and clothing texture. Pearson correlation analysis revealed differential effects of specific food groups (fish, dairy, meat and eggs, bread, and bakeries) on serum vitamin D and HbA1c levels. After adjustment, significant associations were observed for specific food groups, highlighting the dietary influence on both vitamin D status and glycemic control.

**Table 9:** The effect of the different food groups on serum vitamin D and HbA1c levels

Food groups	*r*-value	
	HbA1c	Serum vitamin D levels
Fish	-0.10	0.21**
Dairy	-0.24**	0.28**
Meat and eggs	0.05	0.02
Bread and bakeries	0.18	-0.17

*r*: Pearson Correlation Coefficients for variables: serum vitamin D levels and HbA1c after adjustment of sun exposure, using sunblock and clothing texture.

** Significant correlation. Patients have taken 50,000 IU of vitamin D monthly. HbA1C: glycated hemoglobin; IU: international units.

**Fish intake:** Fish consumption was significantly associated with higher serum vitamin D levels (*r* = 0.21, *P* < 0.01), consistent with (68), which identified fatty fish as a rich dietary source of vitamin D. However, the decrease in HbA1c levels was not significant (*r* = –0.10, *P* > 0.05), and this is likely due to the limited frequency and portion sizes of fish intake in this Jordanian cohort, which may not be sufficient to impact glycemic control significantly.

**Dairy intake:** Dairy consumption was significantly associated with higher serum vitamin D levels (*r* = 0.28, *P* < 0.01) and lower HbA1c levels (*r* = –0.24, *P* < 0.01) in Jordanian adults with type 2 diabetes. These findings align with (69), which revealed that fortified dairy products, such as milk and yogurt, enhance vitamin D status and improve glycemic control, likely due to fortification practices and bioactive compounds that support insulin sensitivity.

**Meat and eggs:** The consumption of meat and egg showed no significant association with serum vitamin D (*r* = 0.02, *P* > 0.05) or HbA1c levels (*r* = 0.05, *P* > 0.05), consistent with their low vitamin D content as reported by (48).

**Bread and bakeries:** The intake of bread and bakery products showed a non-significant inverse correlation with serum vitamin D levels (*r* = –0.17, *P* > 0.05) and a non-significant positive correlation with HbA1c levels (*r* = 0.18, *P* > 0.05). These findings likely reflect the low vitamin D content in these foods and their high carbohydrate content, which may contribute to elevated HbA1c levels when consumed in large quantities.

Overall, the increased consumption of vitamin D-rich foods, particularly fish and dairy, is associated with improved vitamin D status and better glycemic control in Jordanian adults with type 2 diabetes. Fish and dairy intake showed significant positive correlations with serum vitamin D levels (*r* = 0.21 and *r* = 0.28, respectively, *P* < 0.01), highlighting their role as key dietary sources, especially in populations with limited sun exposure. Dairy intake also exhibited a significant inverse correlation with HbA1c levels (*r* = –0.24, *P* < 0.01), suggesting that fortified dairy products may enhance insulin sensitivity through the effects of vitamin D and calcium (69). In contrast, meat, eggs, and bread showed no significant impact on serum vitamin D or HbA1c levels, reflecting their minimal vitamin D content. These findings support dietary recommendations that emphasize fish and fortified dairy products to optimize vitamin D status and glycemic control in patients with type 2 diabetes.

## Discussion

This cross-sectional study of 240 Jordanian adults with T2DM, aged 42–69 years, provides insights into the relationship between the dietary vitamin D intake and metabolic health. The mean dietary vitamin D intake (150 ± 140 IU/day) was substantially lower than in studies that included supplements (29), reflecting the limited reliance on dietary sources in this population, which is compounded by universal monthly vitamin D supplementation of 50,000 IU (~1,667 IU/day). The significant inverse association between the dietary vitamin D intake and HbA1c (*P* < 0.001) aligns with prior research, suggesting that vitamin D enhances insulin secretion and sensitivity via VDRs, calcium signaling, and the reduction of proinflammatory cytokines in pancreatic beta cells (9, 11, 14, 33). A recent meta-analysis reported a 10–15% reduction in T2DM progression with vitamin D supplementation, supporting its role in glycemic control (11, 14). Particularly, dairy intake, including fortified products like yogurt, was associated with lower HbA1c levels (*r* = –0.24, *P* < 0.01), consistent with studies linking fortified dairy to improved insulin sensitivity through VDR activation and modulation of the AMP-activated protein kinase (AMPK) pathway (66, 70). Fish consumption increased serum 25(OH)D levels (*r* = 0.21, *P* < 0.01) but showed no significant effect on HbA1c, likely due to infrequent consumption in this cohort.

Sociodemographic factors, such as higher education and income, were associated with improved serum 25(OH)D levels, likely reflecting better access to vitamin D-rich foods and greater health literacy regarding the benefits of sun exposure (45, 46). These findings are particularly relevant in the Middle East and North Africa (MENA) region, where cultural practices such as head covering limit sun exposure, contributing to widespread vitamin D deficiency (17). The lack of association between the age and serum vitamin D levels (*P* = 0.44) contrasts with findings in non-diabetic populations (43), which may be attributed to uniform statin use or monthly supplementation, potentially stabilizing serum levels across age groups (44). The high prevalence of obesity (57.5%) and overweight (30.0%) aligns with T2DM risk factors. The association between the higher vitamin D intake and lower BMI (*P* = 0.02) supports mechanistic studies linking vitamin D to adipocyte apoptosis and reduced insulin resistance via calcium-mediated pathways (39, 58).

The lack of association between meal frequency and the vitamin D intake (*P* = 0.26) likely reflects regional dietary patterns, where meals predominantly feature foods low in vitamin D, such as rice or legumes, rather than fortified dairy or fish. This suggests that increasing meal frequency alone may not be sufficient to enhance vitamin D intake without prioritizing nutrient-dense sources. The modest sample size (*n* = 240) may have limited the power to detect weaker associations, underscoring the need for larger cohorts and detailed dietary assessments in future studies.

Inconsistencies in vitamin D fortification, particularly in powdered milk and pita bread, highlight gaps in Jordan’s fortification programs. These discrepancies may stem from manufacturing challenges, such as uneven distribution of vitamin D during processing or degradation due to improper storage conditions (e.g. exposure to heat or light). Flour Fortification Initiative (FFI) (60) reported similar nutrient losses in fortified foods under high temperatures or prolonged shelf life in low-resource settings. Additionally, variability in regulatory oversight across regions may lead to inconsistent fortification levels, undermining the reliability of fortified foods as a public health strategy. These issues likely contribute to the suboptimal vitamin D status observed (mean serum 25(OH)D: 22.7 ng/mL) in populations reliant on such products. Enhanced quality control measures and standardized fortification protocols are essential to ensure consistent vitamin D delivery, particularly in the MENA region, where deficiency is prevalent (17).

Sun exposure significantly influenced serum 25(OH)D levels (*P* < 0.001), cultural dress practices, such as wearing hijab or niqab, and likely reduced cutaneous vitamin D synthesis, particularly among women (15, 22–28). This study found no evidence that women with higher coverage compensated for this by increasing their dietary vitamin D intake, indicating a potential double burden of limited sun exposure and low dietary intake. This interaction underscores the importance of targeted dietary interventions in populations with culturally driven limitations on sun exposure, as dietary vitamin D supplementation could help mitigate deficiency risks in these groups.

The universal administration of 50,000 IU monthly vitamin D supplements likely elevated serum 25(OH)D levels across participants, potentially masking the impact of dietary vitamin D intake (11). The pharmacokinetics of monthly dosing, characterized by rapid serum 25(OH)D spikes followed by gradual declines, may obscure the subtler effects of dietary changes (11). Despite this, the association between the dietary vitamin D (e.g. from dairy) and lower HbA1c suggests dietary sources may exert independent metabolic benefits through consistent, low-dose exposure. These findings indicate that dietary associations may be less pronounced in supplemented populations, thereby limiting the generalizability of the findings to settings with less common supplementation practices. Additionally, vitamin D’s potential to reduce T2DM complications, such as neuropathy, further supports its therapeutic role (58). Future studies should explore lower-dose regimens or stratify analyses by supplementation status to isolate dietary contributions and assess broader clinical impacts.

## Limitations

This study provides valuable insights into the relationship between the dietary vitamin D intake and glycemic control in Jordanian adults with type 2 diabetes; however, several limitations should be considered when interpreting the findings.

First, the cross-sectional design precludes establishing causality between the dietary vitamin D intake and glycemic outcomes (e.g. HbA1c levels). Longitudinal or interventional studies are needed to confirm whether increasing dietary vitamin D intake directly improves glycemic control.

Second, dietary data were collected using a FFQ, which relies on self-reported information and is susceptible to recall bias, potentially leading to under- or overestimation of vitamin D intake. Although the FFQ was validated, inaccuracies in portion size estimation or frequency reporting may have affected the precision of dietary intake estimates.

Third, the study did not account for seasonal variations in sun exposure, a critical determinant of cutaneous vitamin D synthesis. Jordan’s climate, with significant sunlight availability, may influence serum 25(OH)D levels differently across seasons, potentially confounding the observed associations between the dietary intake and vitamin D status.

Fourth, the convenience sampling method, while practical, may introduce selection bias, as participants were recruited from government hospitals and may not fully represent the broader Jordanian population with type 2 diabetes, particularly those in private healthcare settings or rural areas with different dietary and lifestyle patterns.

Fifth, all participants received a standard 50,000 IU monthly vitamin D supplement (~1,666 IU/day), which was accounted for in statistical analyses but may have masked the actual contribution of dietary vitamin D to serum levels and glycemic outcomes. The uniform supplementation limits the ability to isolate dietary effects, as supplemental vitamin D likely contributed significantly to serum 25(OH)D levels.

Sixth, this study did not assess genetic factors, such as polymorphisms in VDR genes, which may influence vitamin D metabolism and its effects on insulin sensitivity.

Finally, the sample size (*n* = 240), while adequate for detecting moderate associations, may have been underpowered to detect weaker associations, particularly for non-significant findings (e.g. meal frequency and vitamin D intake). Future studies with larger, more diverse samples and detailed genetic and seasonal data are warranted to address these limitations and provide a more comprehensive understanding of the role of dietary vitamin D in the management of type 2 diabetes.

## Conclusion & future recommendations

This cross-sectional study highlights the potential role of dietary vitamin D in supporting metabolic health. Higher dietary vitamin D intake, primarily from fish and dairy, was positively associated with serum 25(OH)D levels (mean: 22.7 ng/mL) and inversely affected HbA1c (*P* < 0.001), suggesting improved glycemic control. Dairy consumption was particularly linked to lower HbA1c levels, potentially through vitamin D’s role in enhancing insulin sensitivity via VDRs, calcium signaling, and inflammation reduction (33, 71). However, mean dietary vitamin D intake was low (150 ± 140 IU/day), with canned tuna, yogurt, pita bread, and non-fortified UHT milk as the primary sources. Inconsistent fortification in foods such as powdered milk and pita bread highlights gaps in Jordan’s fortification programs, contributing to suboptimal vitamin D status, particularly in the MENA region, where deficiency is prevalent (17).

Sociodemographic factors, including higher education and income, were associated with better vitamin D status, likely due to increased access to nutrient-rich foods and health literacy (45). Sun exposure has significantly influenced serum 25(OH)D levels, though cultural practices of limiting exposure, particularly among women, highlight the importance of dietary sources (15, 17). The universal 50,000 IU monthly vitamin D supplementation likely dominated serum levels, complicating the isolation of dietary effects, yet the observed dietary associations suggest independent benefits (11). Additionally, vitamin D may reduce T2DM complications such as neuropathy, warranting further exploration (58).

These findings support the modifications of food fortification programs, particularly for dairy and staples like pita bread, and promote the consumption of vitamin D-rich foods (e.g. fish and fortified dairy) alongside culturally sensitive sun exposure recommendations. The cross-sectional design and uniform supplementation limit causal inferences, necessitating further research to confirm these associations.

RCTs are recommended to evaluate the efficacy of fortified food consumption (e.g. fortified dairy or pita bread) on serum 25(OH)D and HbA1c levels in Jordanian patients with T2DM, building on evidence from supplementation trials (11, 14). Dietary counseling interventions promoting regular fish and dairy intake could also be assessed for their impact on vitamin D status and glycemic outcomes. Exploring genetic factors, such as VDR polymorphisms, may clarify variability in vitamin D metabolism and response to dietary intake, as these polymorphisms influence receptor activity and insulin sensitivity (71). Longitudinal studies are needed to establish causality, addressing the limitations of the cross-sectional design. Targeted public health strategies, including initiatives such as enhanced fortification protocols, promotion of vitamin D-rich foods, and safe sun exposure guidelines, could address the dual burden of T2DM and vitamin D deficiency in Jordan, thereby improving health outcomes and potentially reducing complications, such as neuropathy (58).
